# Editorial: Prevention and treatment of urolithiasis: innovation and novel techniques

**DOI:** 10.3389/fsurg.2026.1785032

**Published:** 2026-02-27

**Authors:** Steffi Kar Kei Yuen, Bo Xiao, Bhaskar Somani

**Affiliations:** 1 S.H. Ho Urology Centre, Department of Surgery, The Chinese University of Hong Kong, Hong Kong, China; 2 European Association of Urology Section of Endourology (ESEUT), Arnhem, Netherlands; 3 Department of Urology, Research Center for Urinary Disease, School of Clinical Medicine, Beijing Tsinghua Changgung Hospital, Tsinghua University, Beijing, China; 4 Department of Urology, University Hospital Southampton, NHS Trust, Southampton, United Kingdom

**Keywords:** diagnosis, nephrolithiasis, PCNL, percutaneous nephrolithotomy, prevention, RIRS- retrograde intrarenal surgery

## Introduction

The global burden of urolithiasis is substantial and growing, representing a significant public health challenge with profound implications for patient quality of life (QoL) and healthcare systems (1, 2). Despite its considerable clinical and societal burden, urolithiasis remains under-recognized, and under-researched. Greater awareness and investments in innovation, research, and patient education are urgently warranted.

This urolithiasis landscape demands a dual, synergistic focus: enhancing the efficiency and safety of stone removal, and implementing more effective, personalized strategies for recurrence prevention (3). This aligns with the core tenet that the ability to efficiently clear fragments is what ultimately determines procedural success, a challenge directly addressed by advancements in suction technology (4). This series of “*Prevention and Treatment of Urolithiasis: Innovation and Novel Techniques*” bring together fifteen pioneering articles that collectively advance these aims, pushing the boundaries of diagnostic prediction, metabolic understanding, surgical technique, and perioperative care. This research series highlights three converging trends: (1) personalization of screening and prevention, (2) suction-related endourology technology, and (3) predictive analytics guiding procedural choice and perioperative risk.

The imperative for this work is underscored by contemporary epidemiological data. The study “*Trends in the burden of urolithiasis in China: an analysis from the global burden of disease study 2021*” adds onto the existing data a crucial macro-perspective, revealing not only the sheer scale of the disease but also evolving demographic patterns that must inform resource allocation and preventative strategies (5).

True progress in urolithiasis begins with a shift from reactive treatment to proactive, individualized prevention. The cornerstone of this paradigm is a deeper understanding of etiology. The article “*Genetics of kidney stones and the role of genetic testing in prevention: a guide for urologists*” elegantly bridges the gap between molecular science and clinical practice, integrating genetic counseling and testing into the standard metabolic workup for high-risk or early-onset stone formers can unveil monogenic causes allowing for targeted therapy and family screening. This represents the vanguard of personalized medicine in nephrolithiasis (6, 7). Simultaneously, modifiable risk factors remain paramount. The “*Age-stratified analysis of the BMI-kidney stone relationship: findings from a national cross-sectional study*” offers nuanced insights, demonstrating that the obesity-stone risk association is not uniform across the lifespan, thereby refining targeted preventative counseling.

On the therapeutic front, the latest recurrent themes are suction and miniaturized equipments, maximizing efficacy while minimizing morbidity. Suction is arguably the final piece of the puzzle in endourological stone management, facilitating superior stone clearance, improved stone-free rates, and enhanced procedural safety by maintaining lower intrarenal pressures and reducing infectious risks (8–10).

The evolution of percutaneous nephrolithotomy (PCNL), the gold standard for large renal stones, is particularly well-documented (11). We see a move towards optimizing outcomes through better preoperative planning, as exemplified by the “*B.T.C.H. nephrolithometry score: a novel scoring system to predict stone-free rate and complexity for ultrasound-guided percutaneous nephrolithotomy*”. This tool aims to provide surgeons with a reliable, imaging-based metric to tailor their approach. Concurrently, the trend towards minimizing tract size and improving patient comfort is clear (12). As previously demonstrated by the randomized controlled trial by Zeng et al (13), “*Innovations in kidney stone management: mini-PCNL for staghorn calculi in resource-limited settings*” again proved that complex stone burdens can be effectively managed with mini PCNL, reducing bleeding risks and enhancing recovery. The integration of suction in PCNL is a key part of this evolution, contributing to improved outcomes. “*Prospective study of the efficacy of PCNL under local anesthesia based on the ERAS concept*”, challenges traditional anesthesia dogma, demonstrating PCNL under local anesthesia is a feasible, safe, and effective option with significant benefits in postoperative recovery and cost-effectiveness.

Technical ingenuity is further highlighted in approaches to complex anatomy and stone configurations by works “*Needle nephroscope combined with ureteroscope via a single standard percutaneous nephrolithotomy channel for the treatment of complex non-obstructing renal stones*” and “*Percutaneous nephrolithotomy for renal stones combined with laser endoscopy for ipsilateral renal cysts: a case report and literature review*”. “*Retrograde intrarenal surgery in the prone split-leg position for female upper urinary tract stones: a preliminary study of 16 cases*” explores an alternative patient positioning that may offer ergonomic advantages in selected cases.

Flexible ureteroscopy continues to see pivotal advancements with the introduction of flexible and navigable suction ureteral access sheaths (FANS) (8, 9, 14), miniaturized ureteroscopes with integrated functions (15). While “*Paired analysis of flexible and navigable suction ureteral access sheath vs. conventional ureteral access sheath, both combined with needle-perc assisted endoscopic surgery, for the treatment of <2 cm lower calyceal stones with unfavorable anatomy*” still showed the role for both FANS flexible ureteroscopy (fURS) and percutaneous approach, the latest RCT argues that even in 2–3 cm renal stones with larger volume (16), fURS is a viable alternative to mini PCNL with non inferior stone-free rates, lower bleeding risks, shorter hospitalization and superior QoL.

The systematic review “*Risk factors for urosepsis following ureteroscopic lithotripsy: a systematic review and meta-analysis*” provides an evidence-based consolidation of risk factors, a vital guide for preoperative mitigation. Building on this, the “*Nomogram and scoring system for preoperative prediction of the risk of systemic inflammatory response syndrome in one-stage flexible ureteroscopy lithotripsy*” delivers a practical clinical tool, moving from identification to quantifiable prediction of postoperative inflammatory risk.

The quest for surgical efficiency and clearance is also evident in the ureteral stone domain. The “*Comparison of flexible ureteroscopy with flexible and navigable suction ureteral access sheath and mini-percutaneous nephrolithotripsy for the treatment of impacted upper ureteral stones*” directly compares retrograde and antegrade approaches for a difficult clinical scenario, aiding in surgical decision-making. Furthermore, the analysis on “*Integration of minimally invasive techniques and interventional therapy: application of percutaneous nephrolithotomy in patients with upper urinary tract stones and an analysis of risk factors for postoperative bleeding*” reminds us that even as techniques miniaturize, meticulous attention to hemostasis and patient selection is irreplaceable.

Finally, this topic wisely questions longstanding surgical dogmas. The provocative study “*Is negative urine culture necessary for PCNL safety? Experience from a large-volume stone center*” confronts a core tenet of preoperative preparation. While guidelines emphasize the importance of positive preoperative urine culture and antibiotic treatments (6, 17–21), findings from this study reported PCNL performed under controlled fluid dynamics environment is still safe and feasible in patients with positive urine culture which was treated empirically with antibiotics, and a repeat sterile urine culture is not deemed necessary. This highlights the critical role of intrarenal pressure regulation (22–24)—a key rationale for suction use—in mitigating infectious risks regardless of preoperative culture status.

In conclusion, the collective scholarship presented here paints a picture of a dynamic and innovative field. The trajectory is clear: towards prevention grounded in genetics and personalized medicine, and towards treatment that is ever more precise, minimally morbid, efficient, and guided by robust predictive analytics. Suction technology is increasingly positioned to transition from an adjunct to an integral component of modern endourological stone surgery, reshaping our approach by committing to more complete stone clearance and improved outcomes. The innovations detailed—from novel suction devices and hybrid techniques to genetic guides and predictive nomograms—are not merely incremental. They represent meaningful strides in our enduring mission to alleviate the burden of urolithiasis. Future research priorities should include prospective validation of these predictive tools, standardized reporting of endourology outcomes, and long-term outcomes of personalized prevention strategies. By embracing this synergy of prevention and technological refinement, urologists can offer care that is not only more effective but also more compassionate, restoring patients to health with ever-greater speed and safety.
